# Beyond sleepless nights: Unraveling the complexity of alexithymia and suicide risk among university students

**DOI:** 10.1002/brb3.3476

**Published:** 2024-04-15

**Authors:** Ahmad Daghigh

**Affiliations:** ^1^ Department of Brain, Mind, and Education Institute for Cognitive Science Studies Tehran Iran

**Keywords:** alexithymia, insomnia, Persian sample, suicide behavior, university students

## Abstract

**Background:**

There is a solid relationship between alexithymia and suicide risk. Nonetheless, the specific impact of alexithymia's distinct subscales on suicide risk has received the attention it deserves. This article presents a comprehensive exploration of suicide risk among university students, focusing on the interconnections among alexithymia, insomnia, and suicidal behavior. Three components of alexithymia including difficulties in describing emotions or feelings (DDF), difficulties in identifying emotions or feelings (DIF), and the externally oriented thinking were considered.

**Methods:**

The study involved 208 participants from a Persian university sample, examining the significance of incorporating both alexithymia and insomnia in suicide risk assessment and intervention planning. Insomnia was positioned as a pivotal mediator. A secure electronic link in the Telegram application was employed to collect the data. Both linear and nonlinear prediction models were used to explore potential associations among alexithymia, insomnia, and suicide risk.

**Results:**

The study revealed substantial positive correlations between alexithymia and suicide risk, as well as between insomnia and suicide risk. Additionally, specific components of alexithymia exhibited noteworthy links to suicide risk. The inclusion of insomnia scores in suicide risk predictions is critical, as it greatly enhances the precision of risk assessments and facilitates the design of targeted and effective therapeutic interventions. The association between alexithymia and suicide risk showed a significant relationship (*r* = .29, *p* < .01). Moreover, a significant correlation was observed between alexithymia and insomnia (*r* = .32, *p* < .01). Additionally, insomnia exhibited a significant positive correlation with suicide (*r* = .35, *p* < .01). Interestingly, DDF and DIF showed positive correlations with suicide (*r* = .28, *p* < .01; *r* = .33, *p* < .01).

**Conclusion:**

The findings carry profound implications for suicide prevention efforts, providing valuable insights to safeguard the well‐being and resilience of university students facing suicide risk challenges.

## Graphical abstract

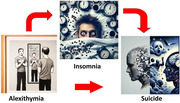

## LIMITATIONS AND FUTURE DIRECTIONS

The present study, while valuable, confronts certain limitations that temper its generalizability to a broader population. The cultural context of the sample, consisting of Persian students with unique life circumstances, prompts questions regarding the transferability of the findings to practical settings. Additionally, the restricted age range of participants limits the extent of extrapolation. Moreover, the adoption of a cross‐sectional design fails to capture potential temporal dynamics in the variables under scrutiny. Employing longitudinal designs would be more advantageous to comprehensively explore temporal changes. Certain subscales within the alexithymia scale exhibited lower reliability. However, given the significance of several variables and the existence of robust and relevant literature, we opted not to exclude them from the assessment procedure. Furthermore, it is crucial to acknowledge that self‐report measures were utilized in our study. Nevertheless, the use of widely recognized and validated scales in this cross‐cultural investigation offers valuable insights into the association among alexithymia, insomnia, and suicide risk, thereby mitigating some of the aforementioned limitations. Further research is warranted to unveil the intricate mechanisms underlying these relationships and to ascertain their causal nature with greater certainty. It is essential to note that emotional symptoms were not controlled for in this study, and future research considering this aspect could enhance overall clarity in understanding these associations.

## 1 INTRODUCTION

The interplay between alexithymia and suicide has garnered significant attention in scientific research, with various investigations exploring their potential association. Alexithymia, characterized by challenges in discerning and expressing emotions, has been postulated to exert a noteworthy influence on suicidal behaviors (Hemming et al., 2019). Nevertheless, it is crucial to highlight that despite the considerable attention given to the link between alexithymia and suicide in scientific research, there is a shortage of comprehensive studies investigating the specific impact of alexithymia's distinct subscales on suicide risk.

Heretofore, research has frequently focused on the overarching concept of alexithymia, neglecting the thorough exploration of its individual components, such as the difficulties in describing emotions or feelings (DDF), difficulties in identifying emotions or feelings (DIF), and the externally oriented thinking (EOT), which warrant more comprehensive investigation.

Moreover, as we strive for a more thorough examination of the individual components of alexithymia in their association with the risk of suicide, it becomes essential to acknowledge the role of insomnia as a potential mediator in this intricate relationship. Insomnia, marked by disruptions in sleep patterns, is a prevalent concern in both clinical and general populations, gaining increasing recognition for its connection to emotional well‐being in scientific literature. While alexithymia, with its elements, has been linked to an increased risk of self‐harm, insomnia may emerge as a crucial piece in understanding the underlying mechanisms that drive this complex association. To the best of the author's knowledge, the role of insomnia as a mediator in the context of the association between the components of alexithymia and suicide risk has not been fully explored in scientific literature.

The necessity for further exploration into the facets of alexithymia, particularly their influence on insomnia and subsequent impact on suicide risk, beckons for examination on multiple fronts. This imperative extends to both clinical and statistical dimensions. Within the clinical realm, it becomes paramount for clinicians to fathom the intricate mechanisms that underlie the emergence of suicidal inclinations within individuals grappling with alexithymia. The pivotal role played by insomnia as an intermediary link in this model stands as a linchpin.

Moreover, discerning that specific components of alexithymia may instigate insomnia as the harbinger of heightened suicide risk equips clinicians with the prescience to identify individuals at an incipient stage of vulnerability. This foresight, in turn, empowers them to implement more proactive interventions, targeting the nexus of insomnia and the intricate fabric of alexithymia's components.

In statistical realm, an analysis that assimilates insomnia as a mediating variable bestows a holistic model. This approach facilitates the scrutiny of both the direct and indirect repercussions of alexithymia's components on the landscape of suicide risk. Through the examination of insomnia's mediating role, researchers can unfurl a clearer panorama of the interplay and intricate connections between these variables.

Various empirical investigations have explored the link between alexithymia and suicide, yielding diverse findings across clinical and general populations. Some studies reported a positive association between alexithymia and suicide ideation and behavior (Álvarez et al., 2021; Bordalo & Carvalho, 2022; Wood et al., 2010). However, complexities emerged as other studies highlighted factors like depression severity and sex influencing this relationship (Bergmans et al., 2020; Hintikka et al., 2004; Sleuwaegen et al., 2017). Moreover, certain investigations found no significant connection between alexithymia and suicide (Xie et al., 2021). Nonetheless, evidence points to alexithymia as a potential contributor to suicidality among individuals with psychiatric disorders (Costa et al., 2020), necessitating further research for a comprehensive understanding and the formulation of targeted suicide prevention strategies.

Advancements in brain sciences, particularly functional neuroimaging studies, have contributed valuable insights to the interplay of alexithymia and suicide (Förster et al., 2020). For instance, Demers et al. (2019) uncovered higher neural activity in response to concealed emotional faces in individuals with elevated alexithymia, particularly in brain regions involved in emotion processing, like the amygdala and insula. This suggests a potential influence of alexithymia on altered emotional processing, contributing to the assessment of suicide risk, particularly in those prone to self‐harm.

Moreover, studies have revealed that heightened levels of DDF, denoting difficulties in articulating emotions, are linked to an elevated risk of suicidal ideation and behaviors (Honkalampi et al., 2018). The struggles in expressing feelings hinder individuals from releasing emotional distress, leading to an accumulation of negative emotions, potentially fostering suicidal thoughts.

Likewise, DIF, representing challenges in recognizing one's own emotions, has been associated with suicide risk. Swart et al. (2009) demonstrated that individuals with elevated DIF scores were more prone to displaying suicidal tendencies. The difficulty in identifying and comprehending emotions may overwhelm individuals and hinder effective coping, heightening susceptibility to suicidal behaviors.

Regarding EOT, investigations have evidenced that a stronger inclination toward EOT, emphasizing external events over internal emotional experiences, is connected to an increased risk of suicide (Marshall et al., 2010). EOT may serve as a defense mechanism against emotional distress, but it may also restrict introspection and emotional processing, potentially contributing to the emergence of suicidal ideation and behaviors.

Studies have consistently attested a significant link between alexithymia and insomnia (Lundh & Broman, 2006). Those with higher levels of alexithymia tend to experience sleep disturbances and insomnia symptoms, including difficulties falling asleep, frequent awakenings, and overall poor sleep quality (Alimoradi et al., 2022). The underlying mechanisms are complex. First, alexithymia's emotional dysregulation can heighten psychological arousal, leading to increased difficulties in initiating and maintaining sleep (Alfasi & Soffer‐Dudek, 2018). The struggle to identify and express emotions may leave unresolved emotional distress, disrupting the sleep–wake cycle (Huntjens et al., 2021). Second, alexithymia often coexists with heightened psychological distress, encompassing anxiety and depression (Fietz et al., 2018). These emotional states can worsen sleep disturbances and contribute to chronic insomnia development (Lyvers et al., 2020). Furthermore, maladaptive coping strategies, such as rumination and worrying, have been linked to alexithymia (Borrill et al., 2009; Mao et al., 2022). These cognitive processes may perpetuate a cycle of sleep‐related anxiety, amplifying difficulties in falling and staying asleep (Pei et al., 2023).

Moreover, a bidirectional relationship is proposed, where alexithymia and insomnia can exacerbate each other (Lumley et al., 2007). Chronic insomnia may trigger emotional dysregulation and reduced emotional awareness, reinforcing alexithymia traits. Research studies have shown that individuals with higher levels of alexithymia, especially elevated DDF scores, are more prone to experiencing sleep disturbances and insomnia (Kano et al., 2003). Additionally, DIF, representing difficulties in identifying one's own emotions, has been linked to insomnia (Montoro et al., 2016). Concerning EOT, studies have demonstrated that a greater inclination toward EOT, where individuals focus on external events rather than their internal emotional experiences, is associated with insomnia (Lee & Lee, 2023). This cognitive style may lead to heightened preoccupation with external stressors, making it difficult for individuals to disengage from worrisome thoughts and attain restful sleep (Greene et al., 2020).

The scientific literature consistently underscores the association between alexithymia and suicide, demonstrating a direct and substantial impact of alexithymia on suicide risk, independently of its relationship with insomnia (De Berardis et al., 2017). Although insomnia has been acknowledged as a potential mediator, research has manifested that alexithymia's influence on suicide risk remains autonomous (Iorga et al., 2017).

Research examining the distinct impact of alexithymia on suicide risk, independent of insomnia, is of utmost importance for profound reasons. Delving into this association beyond potential confounders allows researchers to achieve precise and refined understandings of the direct link between alexithymia and suicide, unraveling the complexities of their interplay (Junus et al., 2023). Furthermore, these empirical inquiries hold practical implications for suicide prevention. If alexithymia emerges as a significant predictor of suicide risk, beyond the influence of insomnia, it underscores the necessity of addressing alexithymic traits in individuals vulnerable to suicidal tendencies (Bakhshesh‐Boroujeni et al., 2021). Tailored interventions targeting emotional awareness and expression in those with alexithymia offer promising avenues for mitigating suicide risk and elevating the efficacy of suicide prevention efforts by mental health professionals.

### 1.1 Statement of the problem

Although insomnia has been recognized as a potential mediator in the relationship between alexithymia and suicidal behaviors, research indicates that alexithymia's impact on suicide risk remains autonomous. Scientific reports investigating the connection between alexithymia and suicide risk in Persian samples have observed similar associations between alexithymia and suicide behaviors. These studies have shown that individuals with higher levels of alexithymia in Persian populations face an elevated risk of suicidal behaviors. However, it remains to be explored whether this linkage holds true beyond the influence of insomnia in Persian samples.

## 2 METHODS

### 2.1 Participants

The sample comprised 208 students randomly selected from three universities in a Persian city, employing simple random sampling. Foreign students were excluded, and only those attending the university were included in the research sample. Among the respondents, 109 (52.4%) were male and 99 (47.6%) were female, with ages ranging from 18 to 32 years old. Notably, 87.1% of the students fell within the 18–25 age group, making young adults the predominant demographic in our research population. Data were collected via a secure electronic link in the Telegram application. The response rate was 68%, and all participants willingly completed the questionnaires without any form of compensation.

### 2.2 Measures

#### 2.2.1 Alexithymia

Alexithymia was assessed using TAS‐20, the 20‐item Toronto Alexithymia Scale, which is a self‐report questionnaire containing 20 items (Bagby et al., 1994). The questions are scored on a five‐point Likert scale from “I strongly disagree (1), I disagree (2), I have no opinion (3), I agree (4), I strongly agree (5),” with total scores ranging from 20 to 100. Moreover, items 4, 5, 10, 18, and 19 are scored in reverse. This questionnaire has three dimensions: DDF (5 questions), DIF (7 questions), and EOT (8 questions). The internal consistency of the alexithymia scale, as assessed through Cronbach's alpha, was determined to be 0.78, denoting a high level of reliability. The scale encompasses nine distinct subscales, with each subscale exhibiting internal consistency as follows: DDF (*α* = .62), DIF (*α* = .85), and EOT (*α* = .50). The average score obtained by the sample amounted to 51.11, with a standard deviation of 9.81. This scale has been validated in Persian samples (Besharat, 2007).

#### 2.2.2 Insomnia

The insomnia severity index (ISI) is a self‐report scale comprising seven items designed to evaluate individuals’ subjective experience of insomnia over the past 2 weeks. Each item is rated on a 0–4 scale, yielding total scores ranging from 0 to 28. Participants were instructed to indicate the frequency of their insomnia symptoms during the previous week, varying from “not at all or less than 1 day” (scored as 0) to “nearly every day for the past 2 weeks” (scored as 4). To assess the internal consistency of the ISI, Cronbach's alpha was employed, resulting in a satisfactory value (*α* = .88). The sample's average score on the ISI was 9.14, with a standard deviation of 6.57. Yazdi et al. (2012) have validated this scale in a Persian sample, ensuring its appropriateness for assessing insomnia severity in this context.

#### 2.2.3 Suicide behavior risk

The SBQ‐R, an updated version of the Suicidal Behaviors Questionnaire, is a comprehensive scale intended to assess suicide risk based on self‐report responses (Daghigh, 2024). Comprising four items, it gauges suicidal ideation, previous suicidal behavior, and self‐assessed likelihood of future suicidal tendencies (Osman et al., 2001). By summing up the scores from these four items, a total index is derived, ranging from 3 to 18. Reliability analyses have consistently demonstrated the SBQ‐R's robust consistency, as evidenced by Cronbach's alpha coefficients of.88 in clinical samples and.87 in nonclinical samples (Osman et al., 2001). In the present sample, the scale displayed satisfactory reliability (*α* = .80). The mean score on the SBQ‐R was 6.46, accompanied by a standard deviation of 3.66, indicating the variability in responses among participants.

#### 2.2.4 Analytic strategy

Data analysis was conducted using SPSS 27. As an exploratory effort, employing correlation tests would reveal overall associations, while regression analyses would discern the individual impacts of alexithymia, its subscales, and insomnia on suicide behavior risk, disentangling their interrelations. Additionally, a quadratic regression was applied to contrast the linear prediction model with the curve model, exploring potential associations among alexithymia, insomnia, and suicide risk in a more nuanced manner.

## 3 RESULTS

To assess the normality assumption, the one‐sample Kolmogorov–Smirnov test was conducted for all variables mentioned earlier. Regarding suicide as the research outcome, the test yielded a test statistic of 0.207 and a *p*‐value less than .001 (*p* < .001). For insomnia, the test statistic amounted to .099 with a *p*‐value also less than .001 (*p* < .001). As for alexithymia, the test statistic was .043, and the associated *p*‐value stood at.200. Having sex as the nominal variable in the correlation test, a decision was made to utilize Spearman's correlation coefficient to achieve a zero‐order association in the first step (see Table 1). The correlation analysis illuminated several significant associations among the variables.

**TABLE 1:** Zero‐order correlations (Spearman) among the variables.

	1	2	3	4	5	6
1. Alexithymia						
2. DDF	.773**					
3. DIF	.892**	.611**				
4. EOT	.462**	.108	.174*			
5. Insomnia	.322**	.217**	.395**	.031		
6. Suicide	.293**	.280**	.331**	.006	.350**	
7. Sex	−.029	−.115	.004	−.004	.039	.061

Abbreviations: DIF, difficulties in identifying emotions or feelings; DDF, difficulties in describing emotions or feelings; EOT, externally oriented thinking.

**Correlation is significant at the.01 level (2‐tailed).

*Correlation is significant at the.05 level (2‐tailed).

The investigation unveiled noteworthy correlations between various constructs. The association between alexithymia and suicide risk displayed a substantial and statistically significant relationship (*r* = .29, *p* < .01). Equally compelling was the significant and robust positive correlation observed between alexithymia and insomnia (*r* = .32, *p* < .01). Additionally, insomnia exhibited a distinct and significant positive correlation with suicide (*r* = .35, *p* < .01).

Delving into the components of alexithymia, it was evident that DDF and DIF showed noteworthy positive correlations with suicide (*r* = .28, *p* < .01; *r* = .33, *p* < .01), whereas the association with EOT did not reach statistical significance. Moreover, in the context of insomnia, DDF and DIF presented meaningful positive correlations (*r* = .21, *p* < .01; *r* = .39, *p* < .01), whereas EOT's linkage remained inconclusive. Finally, sex did not emerge as a significant factor in relation to any of the variables examined in the study, highlighting the impartiality of its influence.

Further, a hierarchical regression analysis was conducted to investigate whether alexithymia predicts suicide behavior risk independent of insomnia. The results of the hierarchical regression analysis have been presented in Table 2.

**TABLE 2:** Prediction of suicide risk by alexithymia and its components above and beyond insomnia.

Regression model	*R* ^2^	*F*	*β*	*t*	*p*
**Alexithymia**	.410	10.249	.083	3.26	.001
**DDF**	.410	10.249	.162	1.969	.050
**DIF**	.410	10.249	.074	1.405	.162
**EOT**	.410	10.249	−.016	–.232	.816
**Insomnia**	.410	10.249	.146	3.84	.001

Abbreviations: DIF, difficulties in identifying emotions or feelings; DDF, difficulties in describing emotions or feelings; EOT, externally oriented thinking.

**FIGURE 1:**
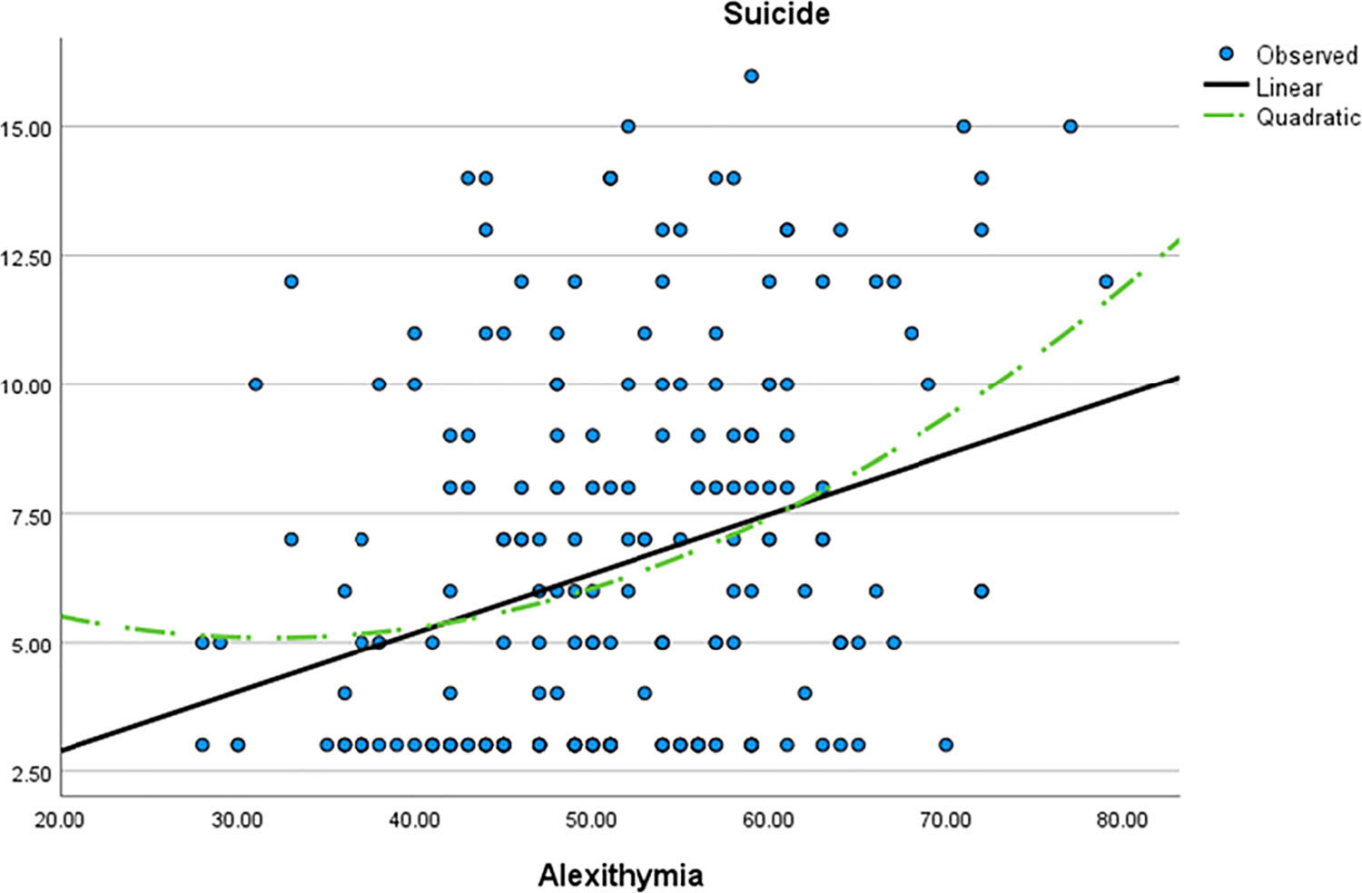
Graphical linear versus curve models in prediction of suicide risk by alexithymia.

In the third step, a nonlinear regression test was exerted to compare a linear model to a curve model in the prediction of suicide risk by alexithymia. The comparison of the linear versus curve estimation has been presented in Table 3 and Figure 1.

**TABLE 3:** Tabular linear versus curve models in prediction of suicide risk by alexithymia.

Regression model	*R* ^2^	*F*	df1	df2	Sig.	Constant
**Linear**	.095	21.54	1	206	.001	.586
**Quadratic**	.106	12.12	2	205	.001	8.08

The analysis explored two regression models in the context of predicting suicide risk based on alexithymia. First, a linear regression model was examined, uncovering an *R*‐squared value of .095, indicating that approximately 9.5% of the variability in suicide risk can be accounted for by the linear relationship between alexithymia and suicide risk. The *F*‐statistic (21.545) established that this linear model is statistically significant (*p* < .001), signifying a meaningful association between alexithymia and suicide risk in a linear model.

Next, a quadratic regression model was introduced, encompassing both linear and quadratic terms of alexithymia. This model attained a higher *R*‐squared value of .106, signifying that approximately 10.6% of the variance in suicide risk can be elucidated by the combined linear and quadratic relationship between alexithymia and suicide risk. The *F*‐statistic (12.124) demonstrated the statistical significance (*p* < .001) of the quadratic regression model, thus highlighting a significant connection between the combined linear and quadratic aspects of alexithymia and suicide risk. The constant term coefficient (8.085) embodies the predicted suicide risk when the value of alexithymia is zero.

**FIGURE 2:**
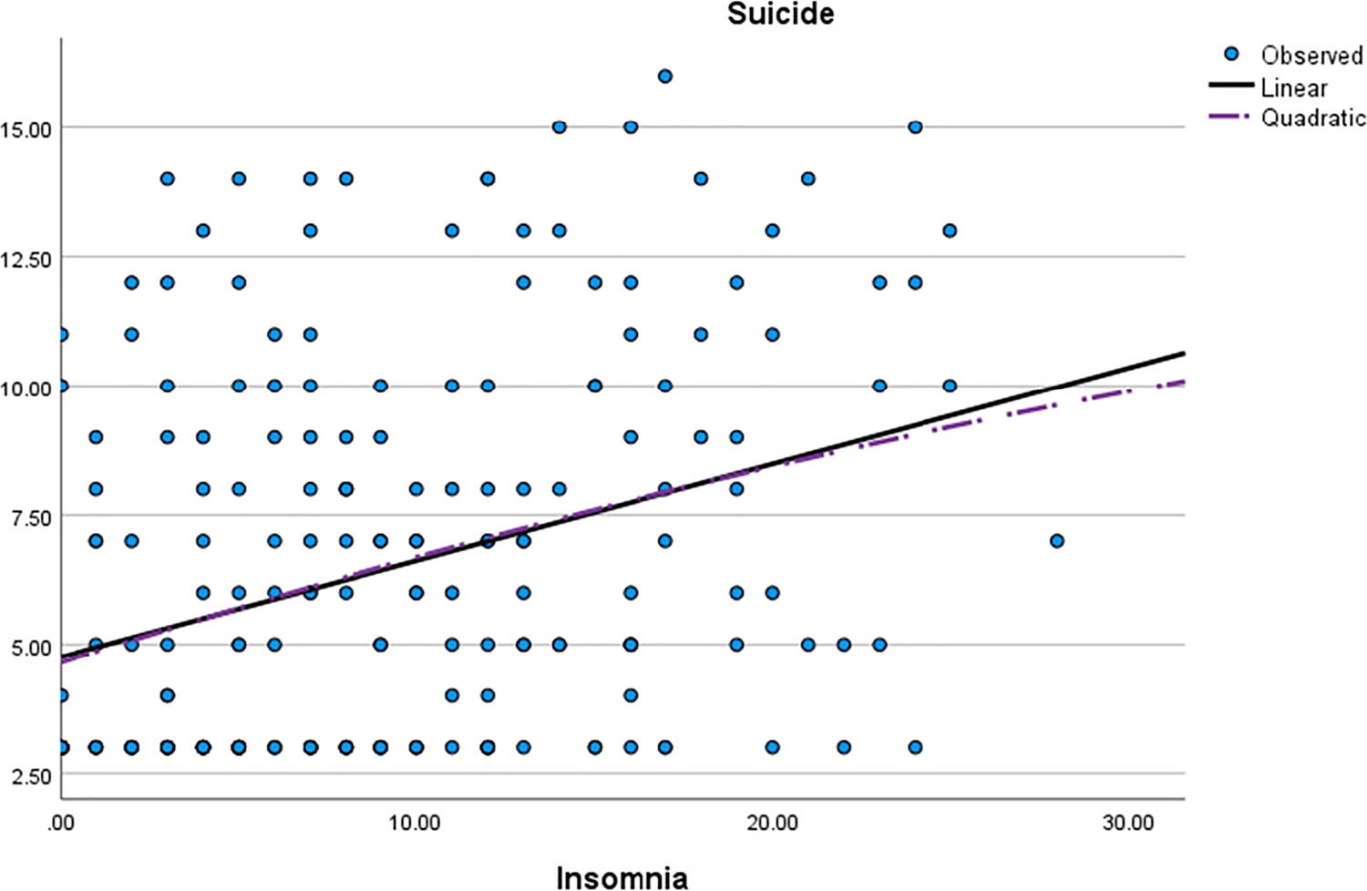
Graphical linear versus curve models in prediction of suicide risk by insomnia.

In the final phase of the analysis, a nonlinear regression examination was performed to contrast the linear and curve models’ predictive capacity concerning suicide risk in relation to insomnia. The comparison between the linear and curve estimations has been documented in Table 4 and graphically depicted in Figure 2.

**TABLE 4:** Tabular linear versus curve models in prediction of suicide risk by insomnia.

Regression model	*R* ^2^	*F*	df1	df2	Sig.	Constant
**Linear**	.112	25.94	1	206	.001	4.75
**Quadratic**	.112	12.95	2	205	.001	4.64

The analysis illustrates that both the linear and quadratic regression models exhibit notable statistical significance in predicting suicide risk based on insomnia (see Figure 2). The *R*‐squared value of.112 for both models indicates that approximately 11.2% of the variability in suicide risk can be accounted for by the relationship between insomnia and suicide risk, encompassing both the linear and quadratic effects. The *F*‐statistics (25.940 for the linear model and 12.954 for the quadratic model) further reinforce the significance of these associations, providing strong evidence of a meaningful relationship between insomnia and suicide risk. This holds true for both the straightforward linear manner and the more nuanced combined linear and quadratic relationship.

## 4 DISCUSSION

This empirical inquiry delved into the intricate relationships among alexithymia, insomnia, and suicide behavior risk disparities within a sample of Persian university students. The variables under investigation exhibited significant deviations from the normal distribution, prompting the utilization of Spearman's correlation coefficient as the analytical tool. The findings yielded a plethora of meaningful associations, elucidating the nuanced interplay between these constructs. The investigation unveiled meaningful and positive correlations among the constructs. Alexithymia exhibited a substantial relationship with suicide risk and displayed a robust positive correlation with insomnia. In turn, insomnia indicated a distinct and significant positive correlation with suicide. Delving into the components of alexithymia, DDF and DIF demonstrated noteworthy positive correlations with suicide, whereas EOT's significance did not reach statistical thresholds. Concerning insomnia, DDF and DIF exhibited meaningful positive correlations, whereas the linkage with EOT remained inconclusive. Sex, on the other hand, exerted no significant impact on the examined variables, signifying its impartiality in the study.

The findings from the hierarchical regression analysis highlighted the contributions of alexithymia and DDF component above and beyond insomnia in predicting suicide risk. However, DIF and EOT did not demonstrate significant associations with suicide risk.

Both the linear and nonlinear models evinced a compelling association between alexithymia and suicide risk, as well as insomnia and suicide risk (see Figures 1 and 2). Comparing Figures 1 and 2 highlights the higher linearity of insomnia in contrast to the U‐shaped plot of alexithymia. This significant finding holds profound clinical weight, offering valuable insights for therapeutic interventions targeting suicide risk management. In certain instances, alexithymic periods and test scores may lack lucidity, posing challenges for psychologists to accurately assess imminent suicide risk. Nevertheless, the incorporation of insomnia scores empowers therapists to make more precise predictions concerning suicide behavior and potential client responses. This augmentation of prediction accuracy can greatly enhance the effectiveness of therapeutic strategies and suicide prevention efforts.

Although these results bear precious implications for enhancing the understanding of the interplay among alexithymia, insomnia, and suicide risk, it is important to exercise caution when generalizing them. As with any study, the specific context of the sample, which consists of Persian university students, should be kept in mind. The broader applicability of these findings to diverse populations and settings should be subject to further investigation. Additionally, the intricacies of alexithymia, its components, and their relationships with suicide risk are multifaceted. Thus, although this study contributes valuable insights, the full comprehension of these intricate connections requires ongoing research and a more comprehensive examination.

The current study's findings align with those of Zhang et al. (2022), who reported similar results in a Chinese sample. Their research demonstrated that both alexithymia and insomnia significantly impact suicidal ideation in adolescents with depression who have experienced childhood maltreatment. Likewise, Liu et al. (2021) investigated the relationship between childhood maltreatment, suicidal ideation in depressed adolescents, and the mediating effect of alexithymia and insomnia.

In a systematic review and meta‐analysis by Hemming et al. (2019), a strong effect size was found for the association between alexithymia and suicide ideation, highlighting a robust link. However, the effect size for the relationship between alexithymia and suicide behavior was comparatively smaller, indicating a weaker connection. Another study by De Berardis et al. (2017) concluded that although alexithymia in individuals who attempted suicide was associated with depressive symptoms, it might not be directly linked to the act of attempting suicide.

Parallel to the present investigation, Bernert et al. (2015) discussed evidence supporting sleep disturbances, particularly insomnia, as a risk factor for suicide. Through a systematic literature review, they established sleep disturbances’ association with increased risk for suicidal behaviors, encompassing suicidal ideation, suicide attempts, and death by suicide.

Mental health professionals in university settings play a critical role in identifying and addressing suicide risk, considering the distinctive challenges faced by this population (Hong et al., 2022). Recognizing the potential impact of alexithymia and insomnia on suicide risk is paramount in this context. Alexithymia's intricate nature, involving difficulties in emotional identification and expression, can pose complexities in an accurate assessment, hindering a comprehensive understanding of suicide risk (Dong et al., 2023). To enhance precision, incorporating insomnia scores as an additional measure allows mental health professionals to gain more insight into underlying emotional struggles masked by alexithymia (Serafini et al., 2020). Combining both alexithymia and insomnia assessments contributes to a more accurate evaluation of suicide risk, facilitating tailored therapeutic strategies for individual needs. Timely identification of suicide risk is crucial, enabling prompt interventions and support systems to avert potential crises (Daghigh, 2024; Daghigh et al., 2019). By considering the interplay between emotional expression difficulties and sleep disturbances, a holistic approach can be devised, addressing the root causes of distress (Weis et al., 2015). This integration of multiple assessment measures empowers mental health professionals to make informed decisions, fostering a proactive and compassionate approach to suicide prevention and mental health promotion within the university environment. Optimizing suicide risk assessments and interventions enables a significant positive impact, bolstering students’ well‐being and resilience as they navigate their academic journey with enhanced emotional support.

## 5 CONCLUSION

This study explores the intricate relationships among alexithymia, its elements, insomnia, and suicide risk. Components of alexithymia include the DDF, DIF, and EOT. Significant associations highlight the strong links between alexithymia and suicide risk, extending to insomnia. Hierarchical regression emphasizes the unique role of alexithymia, particularly DDF in predicting suicide risk independently of insomnia. Both linear and nonlinear models underscore a compelling association, accentuating insomnia's heightened linearity compared to alexithymia. Recognizing the paramount significance of alexithymia and insomnia in shaping suicide risk, this research advocates for timely identification and proactive interventions. Integrating diverse assessments empowers mental health professionals for a nuanced evaluation, fostering tailored therapeutic strategies. The optimized approach to suicide risk assessments supports students’ well‐being throughout their academic pursuits, emphasizing emotional assistance. This study contributes crucial insights into the interplay between emotional expression difficulties and sleep disturbances for suicide prevention among Persian university students. Consequently, the results can be utilized in initiatives designed to prevent suicide among Persian university students and enhance their well‐being and resilience.

## AUTHOR CONTRIBUTIONS

**Ahmad Daghigh**: Conceptualization; methodology; writing—original draft; investigation; visualization; validation; writing—review and editing; formal analysis; software; project administration; data curation; supervision; resources.

## CONFLICT OF INTEREST STATEMENT

The author has no relevant financial or nonfinancial interests to disclose regarding this research, authorship, or publication.

## FUNDING INFORMATION

The author received no financial support or any sort of grant for this research, authorship, or publication.

### CONSENT TO PARTICIPATE

Verbal and in‐person informed consent was obtained from all participants included in the research by the author.

### CONSENT TO PUBLISH

The author thoroughly verifies that human research participants provided informed consent for the publication of this study.

### PEER REVIEW

The peer review history for this article is available at https://publons.com/publon/10.1002/brb3.3476.

## Data Availability

The supporting data for the conclusions drawn in this study can be obtained upon a reasonable request directed to the corresponding author.
